# Maternal Labor Force Participation During the Child’s First Year and Later Separation Anxiety Symptoms

**DOI:** 10.1177/10901981231188137

**Published:** 2023-07-31

**Authors:** Gabrielle Garon-Carrier, Arya Ansari, Rachel Margolis, Caroline Fitzpatrick

**Affiliations:** 1 Université de Sherbrooke, Sherbrooke, Quebec, Canada; 2 The Ohio State University, Columbus, OH, USA; 3 University of Western Ontario, London, Ontario, Canada; 4 University of Johannesburg, Johannesburg, South Africa

**Keywords:** separation anxiety symptoms, labor force participation, household income, longitudinal design

## Abstract

Separation anxiety symptoms are frequent among preschool-aged children, but it is also a possible gateway for diagnosis of separation anxiety disorder. Early maternal employment after childbirth can increase the risk for the development of separation anxiety symptoms. From an economic perspective, however, securing employment is one effective strategy to ensure child well-being. This study investigated how mothers’ participation in the labor force (vs. maternal leave) and the financial state of families when the child was 5 months old was prospectively associated with separation anxiety symptoms. This study is based on 1,295 Canadian families with children assessed longitudinally from 17 months to age 6 on their levels of separation anxiety. Separation anxiety was measured during face-to-face interviews with the mothers. Maternal labor force participation, financial status, and risk factors were measured at 5 months. Results adjusted for propensity scores and for sample weight revealed that children of working mothers, despite having sufficient income (*n* = 245, 18.9%), were at higher risk of separation anxiety during early childhood. In contrast, maternity leave was most beneficial for children’s separation anxiety, whether they were in a family with sufficient income (*n* = 950, 73.4%) or temporary low income (*n* = 100, 7.7%). Children of mothers in maternity leave were at risk of heightened separation anxiety only if they experienced chronic economic hardship. Therefore, maternity leave uptake could help prevent the development of separation anxiety. Providing families with opportunity to care for the baby as their main occupation during this sensitive developmental period could help improve children’s mental health.

Separation anxiety refers to children’s distress from temporary separation from their primary caregiver. During infancy and early childhood, separation anxiety symptoms are developmentally appropriate. It normally manifests between 6 and 12 months of age, after creating a secure attachment with a caregiver, peaks by the age of 3 years, and eventually extinguishes as the child continues to develop cognitively (Vaughan et al., 2017). Yet, early separation anxiety symptoms can also contribute to dysfunctional exaggerated and/or age-inappropriate response to separation in older children, known as separation anxiety disorder (American Psychiatric Association, 2013). Separation anxiety disorder is the most frequent anxiety disorder diagnosed among children (Bufferd et al., 2011, 2019). It accounts for up to 50% of clinical referrals (Cartwright-Hatton et al., 2006) and has a 4% to 8% prevalence rate (Copeland et al., 2014; Ginsburg et al., 2014). Large-scale epidemiological studies have reported 6 years as the age of onset for separation anxiety disorder (Shear et al., 2006). However, recent work indicates that separation anxiety disorder is also common among preschool-aged children (Bufferd et al., 2019; Franz et al., 2013). While it can be transient and remit spontaneously (Ginsburg et al., 2014), it can also lead to poor mental and physical health outcomes in adolescence and adulthood if triggered by life stresses (Battaglia, 2015), including excessive worry and distress in social settings, sleep issues, mood disorders, and alcohol dependence (Vaughan et al., 2017).

According to the attachment theory (Ainsworth, 1989; Bowlby, 1973), the primary caregiver employment early after childbirth may represent a risk for children’s development of separation anxiety symptoms. This is believed to be the case because caregiver employment early after childbirth results in frequent caregiver–child separations and can threaten the establishment of secure attachment and maternal sensitivity. These issues are particularly salient during the early years when young children rely on physical proximity with caregivers as a means of developing attachment. As such, returning to work early after childbirth might compromise the quality of parent–child interactions (Leibowitz, 2005), leading to higher risk of child separation anxiety symptoms. This hypothesis has been supported with studies showing lower sensitivity among full-time working mothers in comparison to part-time working mothers (Brooks-Gunn et al., 2010). Studies show lower levels of maternal sensitivity toward the child when the child spent more time in childcare (National Institute of Child Health and Human Development [NICHD], 2006) and that more than 20 hr/week of childcare attendance at preschool age put a child at risk of developing insecure or disorganized attachments (Quan et al., 2013). In contrast, maternity leave uptake has been directly associated with the quality of mother–child interactions and indirectly linked to attachment security (Plotka & Busch-Rossnagel, 2018). In addition to altering parenting quality and attachment, early maternal employment may contribute to children’s anxiety through increased maternal stress given the demands of employment and parenthood (Costa et al., 2006).

From an economic perspective, however, employment after childbirth may bring economic and social resources to the family, which can benefit children (Becker, 2009). In support of this perspective, a randomized controlled trial revealed that providing mothers with a monthly unconditional cash gift of US$333 resulted in greater infant cerebral activity compared with infants whose mothers received US$20 (Troller-Renfree et al., 2022). The socioeconomic status and levels of maternal sensitivity were also found to be strong predictors of children’s socioemotional development than the amount of time spent in childcare (National Institute of Child Health and Human Development, Early Child Care Research Network, 2003). In addition, the experience of economic disadvantage during early childhood has been associated with higher levels of separation anxiety (Bufferd et al., 2019). Children of low-income families are also more exposed to stressful life situations (Reiss et al., 2019), family dysfunction (Ginsburg et al., 2014), and maternal depression (Goyal et al., 2010), which have been associated with early-onset and more severe internalized/anxious problems. Possible risk factors, such as parenting, can be mechanisms that connect negative familial atmosphere and children’s psychopathology, suggesting the need to control for such family/parenting factors.

Despite the evidence of increased risk of child separation anxiety symptoms among poor families, one previous study conducted in Canada linked maternal employment status at 17 months, but not low socioeconomic status, with higher levels of separation anxiety (Battaglia et al., 2016). This suggests that maternal participation in the labor force may pose risks to the development of separation anxiety, independent of socioeconomic status. The current study examines how mother’s participating in the labor force (working vs. maternity leave) and the financial state of families (sufficient vs. low income) are associated with child separation anxiety symptoms up to age 6. It covers the preschool-aged period, a crucial developmental period where heightened level of separation anxiety manifestations is a possible gateway for diagnosis of separation anxiety disorder at age 6. The attachment hypothesis suggests that children of caregiving mothers (with low income or sufficient income) during the first year of child’s life will have the lowest levels of separation anxiety. In contrast, the economic hypothesis rather suggests that children of working mothers will have lower levels of separation anxiety, in comparison to children of caregiving mothers with low income.

## Method

Participants were from the Quebec Longitudinal Study of Child Development (QLSCD), a longitudinal population study of singletons aimed at understanding the impact of early experiences on later academic and social success. Families were recruited through the Quebec Master Birth Registry of the Ministry of Health and Social Services to be representative of children born in 1997–1998 in Quebec, Canada. For practical reasons, data were not collected on children living on Cree or Inuit territories, in Indian reserves, and in northern Quebec. A three-stage sampling design based on living area and birth rate was used. All selected infants were born after October 1, 1997, to ensure that they entered school the same year. Families were excluded if mothers could not speak French or English and if babies were born before 24 weeks or after 42 weeks of gestation.

A sample of 2,940 families with newborns was initially identified. Selected families that could be located (*N* = 2,675) were approached by mail and phone. Of those, 2,223 families were first visited when the child was 5 months old (83%), and 2,120 (79% response rate) were followed longitudinally and regularly assessed. None of the participating mothers had multiple births. Ethics approval was obtained from the Direction Santé Québec of the Institut de la statistique du Québec and the Faculty of Medicine of the Université de Montréal. The respondent provided consent and voluntarily responded to this survey. The analytic sample for this study included families for whom information was available about the household income and the maternal labor force participation prior to pregnancy and when the child was 5 months old (*N* = 1,295). The study focused on child separation anxiety data collected when children were 17 months (*n* = 1,265), 29 months (*n* = 1,249), 41 months (*n* = 1,230), and then at 4 (*n* = 1,227), 5 (*n* = 1,127), and 6 (*n* = 946) years of age. The average attrition rate was 5.44% per year.

### Measures and Procedure

#### Labor Force Participation

Mothers reported their workforce status prior to birth delivery answering the following question: “Have you worked for pay or profit at any time in the past 12 months (yes/no)?” Only mothers who worked prior to birth delivery were retained in the current study (*n* = 1,553). Mothers also reported their workforce status when the child was 5 months old by answering the following question: “Are you currently working at a job or a business (yes/no)?” Mothers reporting currently working were considered as working mothers (*n* = 395). Those reporting not working were considered in maternity leave (*n* = 1,125).

#### Household Income Status

Mothers also reported the annual household income before tax when the child was age 5 months old. Following the Québec provincial indicator of poverty (Institut de la Statistique du Québec, 2020), household income lower than half of the median household income, once adjusted for the family size and area of residence (ZIP code), were considered as low income (0 = *low income*, 1 = *sufficient income*). This provincial income measure has been compared to the Canadian standard, using the low-income cut-offs measure developed by Statistics Canada (Statistique Canada, 1999), which is the threshold below which a family will likely devote a larger share of its income on the necessities (food, shelter, and clothing) than the average family. Comparison of these two measures provided 99.35% of good classification.

**Figure 1.**
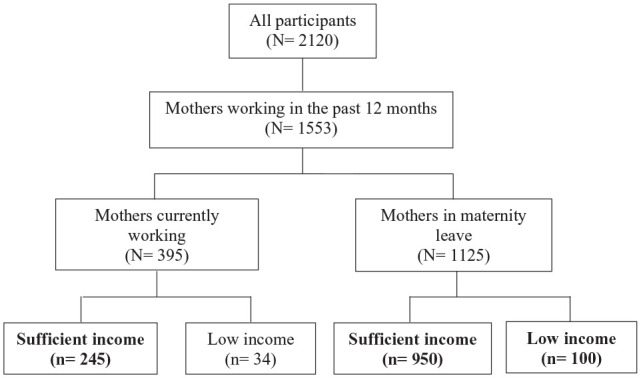
Flowchart of Mothers Based on Their Labor Force Participation and Income When the Child Was 5 Months Old. All the Mothers Worked in the Past 12 Months, Prior to Birth Delivery.

Three groups of mothers were derived from the labor force participation and provincial household income measure (see Figure 1). Non-working mothers when the child was 5 months old with low household income were grouped as “maternity leave with low income” (*n* = 100, 7.7%). Non-working mothers with sufficient household income were categorized as “maternity leave with sufficient income” (*n* = 950, 73.4%). The third group includes “working mothers with sufficient income” (*n* = 245, 18.9%). Only 34 mothers (2.56%) reported working and having a low household income. Because of the small size group, we did not include those participants in our analysis.

Moreover, the mothers also reported their main activity when the child was 5 months old: “What do you consider to be your main activity currently (e.g., caring for family, paid work, full- or part-time student, medical leave/recovering from illness, etc.)?” Mothers in maternity leave (with sufficient and low income) all reported “caring for family” as their main activity while working mothers with sufficient income reported “paid work” as their main activity.

#### Separation Anxiety

Separation anxiety was assessed at 17, 29, and 41 months of age and again at 4, 5, and 6 years of age through parental ratings (0 = *never or rarely*; 1 = *sometimes*; 2 = *often*) of three items from the Child Behavior Checklist (CBCL/1.5–5; Achenbach, 1991), which were collected during face-to-face interviews (e.g., does your child: “react badly when a parent is away,” “not want to sleep alone,” “cling to adults and is too dependent”). These behaviors closely match the manifestations of separation anxiety (i.e., distress related to separation, reluctance to sleep separated from a major attachment figure, fear of being alone or without an attachment figure) and best discriminate children with higher versus lower separation anxiety symptoms (Cooper-Vince et al., 2014).

Principal component analyses of the separation anxiety items at each assessment yielded a single-factor solution accounting for 46.7% to 50.5% of variance, with comparable item loadings 0.74 to 0.78 for “reacts badly when a parent is away,” 0.70 to 0.77 for “clings to adults, too dependent,” and 0.52 to 0.60 for “does not want to sleep alone” (mean α = .80). The separation anxiety factor scores from the single-factor solution were thus used.

### Confounding Variables

Confounding variables were selected as controls for empirical and theoretical reasons (Battaglia et al., 2016). When children were 5 months old, the person most knowledgeable about the child (99.0% were mothers) provided data on maternal education, maternal immigration status, maternal age, paternal age and principal occupation (1 = *family care*, 2 = *working*, 3 = *other* [e.g., studying, medical leave, looking for work]), family composition (single-parent, two-parent, or stepfamily), child sex, Apgar at 5 min after birth (reported in the birth medical registry), and breastfeeding when the child was 5 months old (1 = *yes and I still do*, 2 = *yes but I have since ceased to do so*, 3 = *no, I never did*). Maternal smoking during pregnancy was coded present if the mother had smoked at least one cigarette/day while pregnant, and prenatal alcohol exposure was coded as 0 = *never*, 1 = *having drunk alcohol less than 3 times/month*, and 2 = *having drunk alcohol at least once per week during pregnancy*. History of postpartum depression was coded positive/negative and symptoms of maternal depression in the last week were rated at the 5-month interview with the 12-item version (α = .85) of the Center for Epidemiologic Studies Depression Scale (Poulin et al., 2005). Item responses ranged from 0 (*none*) to 3 (*all the time*), and the total scores were then rescaled on a 10-point scale.

When the child was 5 months old, two dimensions of parenting were reported by the mother with the Parental Cognitions and Conduct Toward the Infant Scale (Boivin et al., 2005): overprotection (e.g., keeping child close most time; 5 items, α = .68) and perceived parental impact (e.g., thinks his or her parenting affects the emotional development of the child; 5 items, α = .71). The mother also reported her perception of the child fussy/difficult temperament (e.g., how much does he or she cry and fuss in general; 7 items, α = .79) with the short form of the Infant Characteristics Questionnaire (Bates et al., 1979), and the levels of positive interactions with the child (e.g., how often do you laugh together; 5 items, α = .81) with the Positive Interaction scale (Boyle & Willms, 2002). Moreover, the emotional and verbal responsivity of the mother (e.g., mother spontaneously vocalizes to the child at least twice during visit; 11 items, α = .87), and the mother involvement toward the child (5 items, α = .86) were measured with the Home Observation for Measurement of the Environment (Bradley & Caldwell, 1984).

Interviews at 17 months also covered child diseases/physical health problems (e.g., allergies, respiratory, cardiovascular), as diagnosed by a health professional and lasting ≥6 months. Externalizing problems were assessed through the CBCL, as defined by a composite score (α = .78) of hyperactivity (Galera et al., 2011) and physical aggression (Côté et al., 2007). Child’s sleep problems were indicated by less than 6 consecutive hours of sleep per night (Touchette et al., 2005).

## Analytical Strategy

The average proportion of missing data across covariates was 2.2%. Considering the low proportion of missing data, missing data were replaced by the mean for continuous variables, the median for ordinal variables, or the mode for categorical variables. The proportion of missing data on child separation anxiety ranged from 2.3% to 27.0%. According to Little’s test, the overall pattern of missingness did not significantly deviate from a pattern of data being missing completely at random (χ^2^ = 78.06, df = 72, *p* = .292). However, a series of *t*-tests revealed that children whose separation anxiety scores were missing tended to be from lower socioeconomic background.

**Figure 2:**
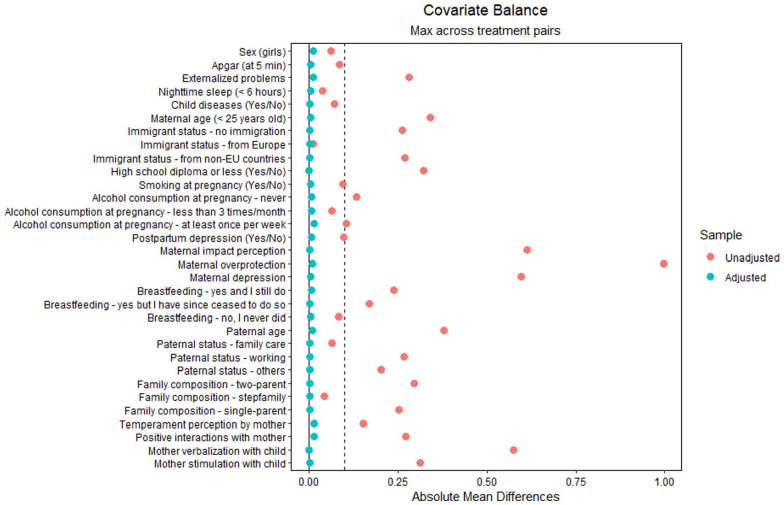
The Average Standardized Mean Difference Between Groups for All the Covariates Used in the Estimation of the Propensity Score Weights (PSW). The Orange Dots Represent the Differences Among Groups on Each Covariate Before Applying the PSWs, and the Blue Dots Represent the Differences After Application of the PSWs.

We performed covariate balancing propensity score (CBPS) weighting to reduce selection bias across the three groups of mothers in R (Imai & Ratkovic, 2014). This method assumes that the household income and maternal workforce status are *not* random and minimizes their associations with the covariates. CBPS performed multinomial regression to test the associations between the covariates and the three groups of mothers and generates a propensity score for each observation. Propensity score estimates the predicted probability of group membership from all observed covariates. Once estimated, we conducted a balancing test to ensure the quality of weighting (Smith & Todd, 2005). As shown in Figure 2, the balancing test showed that all covariates had a standardized mean difference less than |.10| after CBPS, indicating minimal group differences (Pan & Bai, 2015).

To compare the levels of separation anxiety across age and groups, a two-way repeated-measures analysis of variance (ANOVA), 3 (groups) × 6 (time) was performed. This analysis was adjusted for the propensity scores and for sample weight from the QLSCD, which ensure that the sample is representative of the Quebec population. The Greenhouse–Geisser correction was applied when the sphericity assumption was not respected. Post hoc comparisons were corrected with the Bonferroni procedure. Code for this study is available by emailing the corresponding author.

## Results

About 53.0% of working mothers and 62.0% of mothers in maternity leave with sufficient income had an annual income of Can$50,000 or more. In contrast, mothers in maternity leave with low income had Can$29,999 or less per year. Prior to applying CBPS, differences between groups frequently pertained to mothers in maternity leave with low income. Descriptive statistics for each covariate by maternal workforce and household income status are presented in Table 1.

**Table 1.** Child, Maternal, and Family-Wide Factors at Birth and at 5 and 17 Months (*N*= 1,295) Associated With the Maternal Workforce and Income Status When the Child Was 5 Months Old, Prior to Applying CBPS.

	Maternity leave		Working
Child, Maternal, and Family-Wide Factors	Low income (*n* = 100, 7.7%)	Sufficient income (*n* = 950, 73.4%)	Sufficient income (*n* = 245, 18.9%)
Child characteristics			
At birth			
Sex (girls, *n* = 633)	43.0%	49.6%	48.6%
Apgar (at 5 min)^a^	9.37 (0.69)	9.34 (0.80)	9.43 (0.75)
At 5 months			
Difficult temperament of the baby	2.83 (1.73)	2.78 (1.59)	2.49 (1.46)
At 17 months			
Externalized problems^a^	2.75 (1.68)	2.26 (1.61)	2.33 (1.52)
Nighttime sleep (<6 hr, *n* = 73)	10.0%	5.4%	4.9%
Child diseases (*n* = 115)	13.0%	8.1%	10.2%
Maternal characteristics			
At birth			
Maternal age (<25 years old, *n* = 199)	48.0%	11.2%	18.4%
Immigrant status (*n* = 91)	24.0%	6.0%	4.1%
High school diploma or less (*n* = 224)	42.0%	15.7%	13.5%
Alcohol consumption at pregnancy (*n*= 514)	30.0%	40.7%	39.6%
Smoking at pregnancy (*n* = 268)	31.0%	19.8%	20.0%
Postpartum depression (*n* = 384)	23.0%	31.4%	25.7%
At 5 months			
Maternal depression^a^	6.99 (5.63)	4.73 (4.68)	4.25 (4.35)
Breastfeeding (yes I still do, *n* = 447)	34.0%	39.8%	14.3%
Maternal impact perception^a^	7.68 (2.21)	8.71 (1.56)	8.57 (1.64)
Maternal overprotection^a^	5.80 (2.48)	4.43 (2.06)	3.85 (1.92)
Positive interactions with the baby^a^	8.84 (1.13)	9.11 (0.98)	8.90 (1.19)
Verbalization to the baby^a^	6.13 (1.76)	6.89 (1.49)	7.00 (1.35)
Involvement toward the baby^a^	4.26 (2.31)	4.96 (2.27)	4.83 (2.14)
Family-wide factors			
At 5 months			
Paternal age^a^	30.09 (6.06)	32.64 (4.88)	32.38 (5.13)
Paternal working status (working, *n* = 1,164)	67.0%	92.3%	89.8%
Family composition (single parent, *n* = 44)	24.0%	2.0%	0.4%

*Source.* Data are courtesy of the Quebec Institute of Statistics.

*Note.* CBPS = covariate balancing propensity score.

a Continuous variables.

### Separation Anxiety by Maternal Workforce and Income Status

**Figure 3.**
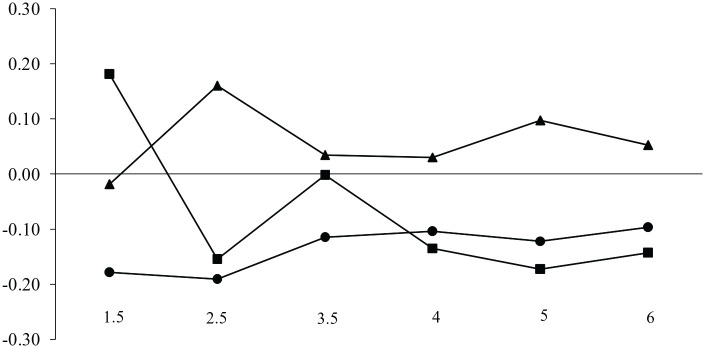
Patterns of Separation Anxiety Based on Factorial Scores: 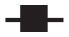 Children of Mothers in Maternity Leave With Low Income (*n* = 100, 7.7%), 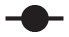 Children of Mothers in Maternity Leave With Sufficient Income (*n* = 950, 73.4%), and 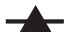 Children of Working Mothers (*n* = 245, 18.9%). *Source.* Data courtesy of the Quebec Institute of Statistics.

Results are shown in Figure 3. A significant group by time interaction was found, *F*(8.88, 15198.41) = 22.51, *p* < .001, η^2^ = .013. Between-group comparisons revealed significant differences on the levels of separation anxiety from 1.5 to 6 years. Planned pairwise comparisons, displayed in Table 2, revealed that children of mothers in maternity leave with sufficient income had significantly lower levels of separation anxiety in comparison to children of working mothers from ages 1.5 to 6 years. They also had lower separation anxiety at 1.5 and 3.5 years in comparison to children of mothers in maternity leave with low income, but revealed no significant difference with this group afterward. In contrast, children of working mothers had the highest levels of separation anxiety at ages 2.5 and from ages 4 to 6 years. In other words, children of mothers in maternity leave with low income had, on most time points, lower levels of separation anxiety symptoms in comparison to children of working mothers and no significant difference with children of mothers in maternity leave with sufficient income.

**Table 2.** Marginal Mean Estimates With 95% Confidence Interval (CI), Adjusted for the Propensity Scores and for Sample Weight.

	Maternity leave		Working
Separation anxiety	Low income	Sufficient income	Sufficient income
	a	b	c
17 months (1.5 years)	0.181 [0.121, 0.240]^b,c^	−0.179 [−0.245, −0.114]^a,c^	−0.019 [−0.078, 0.040]^a,b^
29 months (2.5 years)	−0.155 [−0.215, −0.095]^c^	−0.191 [−0.257, −0.125]^c^	0.160 [0.101, 0.219]^a,b^
41 months (3.5 years)	−0.002 [−0.055, 0.050]^b^	−0.115 [−0.172, −0.057]^a,c^	0.034 [−0.018, 0.086]^b^
4 years	−0.135 [−0.188, −0.082]^c^	−0.104 [−0.163, −0.045]^c^	0.030 [−0.023, 0.082]^a,b^
5 years	−0.173 [−0.226, −0.120]^c^	−0.122 [−0.181, −0.064]^c^	0.097 [0.045, 0.149]^a,b^
6 years	−0.143 [−0.197, 0.090]^c^	−0.097 [−0.155, −0.038]^c^	0.052 [0.000, 0.105]^a,b^

a, b, c Letters indicate significant difference with the groups.

### Sensitivity Analysis

We conducted supplementary analysis to explore the longitudinal contribution of the household income status. The groups of mothers were derived from the labor force participation at 5 months and the provincial income measure when the child was 5 and 17 months old. The results are shown in Supplemental Materials. Children of mothers in maternity leave with sufficient income at 5 and 17 months tended to have the lowest levels of separation anxiety symptoms across time. Children of mothers in maternity leave with low income up to 17 months had the highest levels of separation anxiety at 1.5, 3.5, and 4 years. They had a steep increase in separation anxiety symptoms at ages 3.5 and 4 years followed by a steep decrease in symptoms at ages 5 and 6. This steep increase may possibly reflect impaired coping with new challenging/stressful situations as it coincides with entrance into junior kindergarten program. The significant decrease in separation anxiety symptoms around age 5, however, suggests temporary heightened separation anxiety around 3.5 for these children. Children of working mothers with sufficient income tended to show a steady trajectory of separation anxiety symptoms.

## Discussion

Previous studies have shown evidence that the maternal workforce and household income status may lead to heightened levels of child separation anxiety symptoms during early childhood (Battaglia et al., 2016; Bufferd et al., 2019). According to the attachment theory (Ainsworth, 1989; Bowlby, 1973), early employment could threaten secure mother–child attachment by reducing access to the primary caregiver and increasing the risk for the development of separation anxiety. From an economic perspective, however, securing employment is one effective strategy to ensure child well-being. Using a population-based sample, we explored whether maternal workforce and household income status at 5-month postpartum was associated with children’s development of separation anxiety symptoms through age 6. Taken as a whole, our study shows that after adjusting for confounding variables, children of working mothers were at higher risk of separation anxiety during early childhood. In contrast, maternity leave was most beneficial for children’s separation anxiety, whether they were in a family with sufficient income or *temporary* low income (5 first months of child’s life). Children of mothers in maternity leave were at risk of heightened separation anxiety only if they experienced *chronic* economic hardship.

One previous study showed that socioeconomic hardship predicts mental health disorder onset in childhood, including separation anxiety disorder (McLaughlin et al., 2011). However, this study did not take into account parenting characteristics, including the quality of mother–child interaction, in its prediction. One other study revealed that the length of maternity leave is associated with the quality of mother–child interactions and attachment security (Plotka & Busch-Rossnagel, 2018). Studies examining the effect of leave policy on child developmental outcomes across the life course, however, are sparse and generate conflicting results (Nandi et al., 2018). One Canadian study, which excluded the province of Québec and single-household families, found small improvements in the parental leave program on child language skills, but no associations with child anxiety and externalizing behaviors at 4 and 5 years (Baker & Milligan, 2015). One U.S. study indicated that paid leave was associated with better language outcomes regardless of socioeconomic status and with fewer infant behavior problems for mothers with lower levels of educational attainment (Kozak et al., 2021). According to another study from the United States, returning to work before 3 months after childbirth was associated with increased risk of child externalizing behaviors at age 4 (Berger et al., 2005). Child internalizing/anxious behaviors were not measured in that study. To our knowledge, this study is the first to examine how maternal labor force participation (working vs. maternity leave) and the financial state of the families early after childbirth longitudinally predict separation anxiety symptoms.

### Implications for Social Policy

Parental leave is presumed to benefit attachment between parents and infants and to improve parent and child outcomes by allowing parents to build family relationships and reducing work–family conflicts (Glass & Estes, 1997; Repetti et al., 2009). Our results indicate that mothers in maternity leave while having sufficient income was the most common (73.4%) and optimal condition to prevent separation anxiety symptoms, possibly because it favors mother–child interactions and attachment security (Plotka & Busch-Rossnagel, 2018). Decisions to use the parental leave program partly depend on the length of leave and amount of earning coverage (Slopen, 2020). The 55% wage subsidy in Canada may make it difficult for low-income families to remain out of the workforce (McKay et al., 2016) or to adequately meet necessities, which may contribute to inequity in newborns’ early life experiences (Margolis et al., 2019; Reichelt et al., 2020). In particular, our findings revealed that returning to work prior to 5 months after childbirth or being in maternity leave and experiencing *long-term* economic hardship was an additive risk factor for acute levels of separation anxiety and may signal enlarged risk for future child psychopathology, such as separation anxiety disorder. That is why a minimum of guaranteed paid leave (Schindler-Ruwisch & Eaves, 2022) could prevent mothers from working or experiencing long-term financial precarity and thus could help benefit children’s mental health.

### Limitations and Future Directions

This study should be interpreted in the context of five main limitations. First, the assessment of separation anxiety symptoms was reduced to only three key items and relied on the parents’ perception of the child behaviors, which can introduce measurement bias. However, these items have been shown central by Item Response Theory analyses to the construct and identification of children manifesting separation anxiety (Cooper-Vince et al., 2014). Second, our study did not test how the attachment style of babies contributes to child’s separation anxiety symptoms. It rather controlled for parenting quality and the mother–child interactions, as factors contributing to the development of a secure attachment (Birmingham et al., 2017) and to children’s mental health (Kerns & Brumaritu, 2014). Third, this study did not include a comparison group of low-income working mothers (*n* = 34). In the absence of comparisons with this group, the effect of both the working status and the effect of low household income status on separation anxiety may have been underestimated in the current study. Fourth, the first time-point of data collection was measured in 1998. The maternity leave policy context has changed since then and participating families may also not reflect the current diversity of the Québec population, which could reduce the generalizability of our findings. Nevertheless, we believe these findings can help guide parental leave policies. In the United States, the Family and Medical Leave Act only guarantees up to 12 weeks of unpaid leave, providing short maternity leave and dragging mothers into financial insecurity. In Québec, where this study has been conducted, the maternity leave is up to 18 weeks at 70% of the income. As such, the Québec parental leave policy is likely to reduce the risk of heightened child separation anxiety symptoms. Fifth, this study is limited by the labor force participation (vs. maternity leave) measurement when the child was 5 months old. It would be worthwhile in future studies to explore how the maternal labor force participation 1 year after childbirth, which correspond to the most common return-to-work situation in Quebec (Findlay & Kohen, 2012), associated with separation anxiety symptoms.

## Conclusion

Maternity leave uptake is preferable for the levels of child separation anxiety symptoms during the preschool years in comparison to participating in the labor force, and even more so when the household income is sufficient. However, experiencing chronic financial insecurity seemed to co-occur with more separation anxiety symptoms. Children of mothers in maternity leave were at higher risk of separation anxiety if they experienced chronic economic hardship, suggesting that both maternal working and income status matters in preventing heightened levels of separation anxiety. As such, providing families with the opportunity to care for the baby while having financial security during the first year of a child’s life could benefit children’s long-term mental health and promote healthy child development.

## Supplemental Material

sj-docx-1-heb-10.1177_10901981231188137 – Supplemental material for Maternal Labor Force Participation During the Child’s First Year and Later Separation Anxiety Symptoms Click here for additional data file. Supplemental material, sj-docx-1-heb-10.1177_10901981231188137 for Maternal Labor Force Participation During the Child’s First Year and Later Separation Anxiety Symptoms by Gabrielle Garon-Carrier, Arya Ansari, Rachel Margolis and Caroline Fitzpatrick in Health Education & Behavior
