# Supplementary material for: Maternal Labor Force Participation During the Child’s First Year and Later Separation Anxiety Symptoms
Source: Health Educ Behav. 2023 Jul 31;50(6):792–801. doi: 10.1177/10901981231188137 (PMC10638856; doi:10.1177/10901981231188137)
Supplement: sj-docx-1-heb-10.1177_10901981231188137 – Supplemental material for Maternal Labor Force Participation During the Child’s First Year and Later Separation Anxiety Symptoms [file sj-docx-1-heb-10.1177_10901981231188137.docx]

**Supplementary materials: Results of the sensitivity analysis**

3.5

2.5

***Sensitivity analysis***

Sensitivity analysis conducted with the provincial income measure at both 5 and 17 months old revealed 96% of good classification across time for families with sufficient income, suggesting that most families with sufficient income when the child was 5 months-old were still categorized as having sufficient income when the child was 17 months. However, 30.0% of the mothers in maternity leave with low income when the child was 5 months were not below the threshold of low income anymore when the child was 17 months. As such, we conducted supplementary analysis on groups of mothers derived from the labor force participation at 5 months old and the provincial income measure when the child was 5 and 17 months old. Specifically, mothers changing from one financial status to another from 5 to 17 months (e.g., low to sufficient income, or sufficient to low income) were removed from the analysis.

We conducted a two-way repeated measures analysis of variance (ANOVA), 3 (groups) × 6 (time) where mothers in the maternity leave with low income (*n*= 65, 5.5%) were compared with mothers in the maternity leave with sufficient income (*n*= 898, 75.4%) and working mothers with sufficient income (*n*= 228, 19.1%). Once again, the analysis was adjusted for the propensity scores and for sample weight from the QLSCD. Similar to our previous findings, a significant group by time interaction was found, F(9.15, 12463.59)= 23.09, *p*= .000, η^2^= .017. Children of mothers in maternity leave with sufficient income at 5 and 17 months tended to have the lowest levels of separation anxiety symptoms across time. Children of mothers in maternity leave with low income at 5 and 17 months had the highest levels of separation anxiety at 1.5, 3.5 and 4 years. They had a steep increase of separation anxiety symptoms at ages 3.5 and 4 years followed by a steep decrease of symptoms at ages 5 and 6. In contrast, children of working mothers with sufficient income tended to show a steady trajectory. Results are shown in Table S1 and Figure S1.

**Results of the two-way repeated measures analysis of variance (ANOVA), 3 (groups) × 6 (time) using groups of mothers derived from the labor force participation and provincial income measure when the child was 5 and 17 months old. The analyses excluded children from families that changed from low to sufficient income, or from sufficient to low-income status from 5 to 17 months.**

Table S1. Marginal mean estimates with 95% confidence interval (CI), adjusted for the propensity scores and for sample weight

| Outcome | **Maternity leave** | |  | **Working** |
| --- | --- | --- | --- | --- |
|  | Low-income  (n= 65, 5.5%) | Sufficient income  (n= 898, 75.4%) |  | Sufficient income  (n= 228, 19.1%) |
| *Separation anxiety* | **a** | **b** |  | **c** |
| 1.5 years | .011 [-.064; .085] ^b^ | -.190 [-.250; -.130] ^a,c^ |  | -.025 [-.078; .029] ^b^ |
| 2.5 years | -.097 [-.183; -.011] ^c^ | -.190 [-.259; -.121] ^c^ |  | .149 [.088; .211] ^a,b^ |
| 3.5 years | .145 [.071; .219] ^b^ | -.126 [-.185; -.066] ^a,c^ |  | .034 [-.019; .087] ^b^ |
| 4 years | .293 [.218; .367] ^b,c^ | -.116 [-.176; -.055] ^a,c^ |  | *.*021 [-.033; .074] ^a,b^ |
| 5 years | -.175 [-.249; -.101] ^c^ | -.152 [-.212; -.092] ^c^ |  | .094 [.041; .147] ^a,b^ |
| 6 years | -.257 [-.335; -.180] ^b,c^ | -.100 [-.162; -.037] ^a,c^ |  | .044 [-.011; .099] ^a,b^ |

^a, b, c,^ letters indicate significant difference with the groups

1.5

6

5

4

3.5

2.5

**Figure S1.** Patterns of separation anxiety based on factorial scores: children of mothers in maternal leave with low-income (n= 65, 5.5%), children of mothers in maternity leave with sufficient income (n= 898, 75.4%), children of working mothers (n= 228, 19.1%). Data courtesy of the Quebec Institute of Statistics.
